# Challenges of the Calgary–Cambridge Consultation Guide in Veterinary Multicultural and Multilingual Scenarios and the Role of Veterinary Translators

**DOI:** 10.3390/ani14152270

**Published:** 2024-08-04

**Authors:** Angel Almendros, Paulo V. Steagall, Suen Caesar Lun, Jonathan Speelman, Antonio Giuliano

**Affiliations:** 1 Department of Veterinary Clinical Sciences, Jockey Club College of Veterinary Medicine and Life Sciences, City University of Hong Kong, Hong Kong SAR, China; 2 Veterinary Medical Centre, City University of Hong Kong, Hong Kong SAR, China; 3 Centre of Animal Health and Welfare, City University of Hong Kong, Hong Kong SAR, China; 4 Department of Linguistics and Translation, College of Liberal Arts and Social Sciences, City University of Hong Kong, Hong Kong SAR, China; 5 Advanced Vetcare, Kew, VIC 3101, Australia

**Keywords:** back-translation, Calgary–Cambridge, communication, consultation, multicultural, multilingual, interpreter, veterinary translation

## Abstract

**Simple Summary:**

The success and the satisfaction of clinicians in practice relies heavily on how consultations, communication, and relationships are built with clients. Very knowledgeable and competent veterinarians might not be able to provide their services if the client declines their services based on misperceptions or lack of understanding, potentially resulting in lack of treatments of pets in need. The Calgary–Cambridge Guide for consultations and communication is an important tool taught in most veterinary colleges to help future clinicians improve their communication skills, ultimately benefiting the veterinary profession, clients, and the welfare of their pets. In this commentary we describe challenges in applying these guidelines in multilingual and multicultural scenarios such as Hong Kong. In these scenarios clinicians and clients often need an interpreter, adding complexity to the interaction and communication. Non-verbal communication, where body language plays an important role in showing expressions, empathy, and concerns is not effective or is altered if there is not even eye contact or a translator is not accurately interpreting all these emotions. This commentary analyses the challenges encountered by veterinarians during consultations in multicultural and multilingual centres.

**Abstract:**

The Calgary–Cambridge Guide is a widely recognised framework for teaching communication skills to healthcare professionals that has become a cornerstone of communication training programs in medicine and other healthcare fields. In the context of veterinary medicine, its integration into communication training programs has become an asset improving communication, education, interaction, and quality of service, enhancing the veterinary–client–patient relationship (VCPR). In veterinary medicine, however, a more challenging consultation dynamic involves the veterinarian, the owner, and the animal. The addition of a veterinary assistant that acts as an interpreter or translator is common in Hong Kong where the native language (Cantonese) coexists with English when consultations are led by non-native language speakers. This addition converts this commonly dyadic model into a triadic communication model. The addition of an assistant interpreter influences the way consultations are conducted, how information is conveyed, and how interpersonal cues and empathy are delivered. In this report we depict challenges applying the Calgary–Cambridge Guide in multicultural and multilingual veterinary medical centres in Hong Kong and highlight the role of veterinary supporting staff in these scenarios, specifically veterinary assistant interpreters.

## 1. Introduction 

Clinical competence in healthcare was traditionally focused on application of medical knowledge and disregarded communication, which was not considered a clinical skill. This approach was poorly interactive and communication consisted of information merely being transmitted between parties, ignoring needs, burdens, values, or concerns of clients that could potentially lead to discontent, misunderstanding, poor compliance, and ultimately higher risk of litigation [1]. Poor communication has a negative impact on the practitioner–client or veterinary–client–patient relationship (VCPR), and leads to an overall diminished practice satisfaction [2,3].

**Figure 1:**
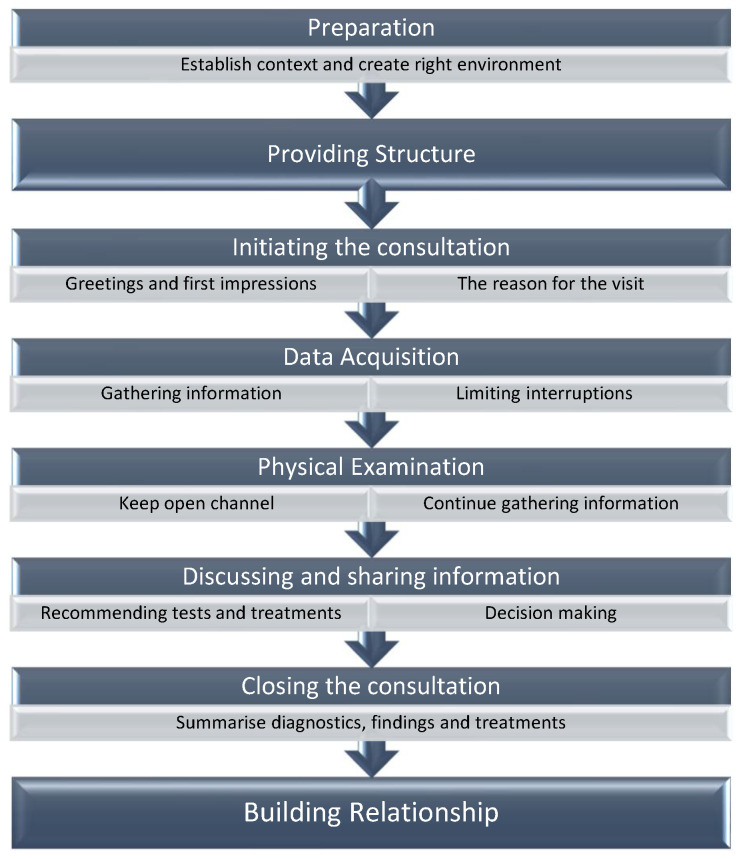
Communication guide taught to undergraduates at City University of Hong Kong based on the adapted model of the Calgary–Cambridge guide by Radford et al., 2006 [6].

To address these shortcomings, communication training emerged, becoming a vital element in healthcare education that is included now in most teaching curricula and provides students and professionals with these essential clinical skills [2,4]. The veterinary community has widely adopted guidelines such as the Calgary–Cambridge Guide (CCG), or modifications of it, as part of their veterinary training [5,6,7,8]. The CCG provides a structured approach to communication, divided into distinct stages that healthcare professionals should master. These stages, seen in Figure 1, are taught at the veterinary college of City University of Hong Kong and include preparation, initiating the session, gathering information, physical examination, explaining and planning, and closing the session while providing structure and building a relationship with clients [5,6]. Within each stage, the guide outlines specific communication skills and behaviours that are essential for effective interactions. 

Consultation structure models, as well as their integration into veterinary communication training programs, aim to improve effectiveness of consultation skills for students and graduates [6]. The knowledge acquired through these guidelines is applied by the clinicians throughout their career, with an iterative component and personal adaptations [9]. These guidelines can be tailored to align also with the specific communication competencies of different specialties, addressing the unique communication skills and behaviours required in different healthcare settings like pharmacy or emergency medicine consultations [10,11]. Within veterinary medicine, modifications of the CCG have been successfully applied to various animal species too [7,12,13]. With the evolution of global online platforms, the role of communities of practice (CoPs) fostering communication skills among veterinarians could help to overcome more specifically some of the challenges encountered in multicultural and multilingual settings [14].

The authors acknowledge the important role and the challenges encountered by supporting staff in communication during consultations in veterinary medicine, suggesting that, similar to what has been completed in other health care disciplines, additional support as well as further research should be conducted [15,16,17]. Additionally, the guidelines taught in veterinary education or other healthcare disciplines, may require further adaptations when applied to multicultural and multilingual centres (MCMLs) due to the introduction of assistant interpreters. In essence, these guidelines are based or better suited for two individuals who are speaking the same language; however, this is not the case at MCMLs where supporting staff, such as veterinary assistant interpreters (VAI), play a crucial role in this triadic communication model. In these cases, communication therefore adopts a triangular model in a three-way conversation with some essential differences that will be addressed in this paper.

## 2. Contextual Framework of Veterinary Consultations in Hong Kong

### 2.1. Institutions

The authors teach and/or practice at the Jockey Club College of Veterinary Medicine and Life Sciences of City University of Hong Kong (JCC), at the Veterinary Medical Centre of City University of Hong Kong (VMC), which acts as a teaching hospital for the JCC, or at the Department of Linguistics and Translation of the same university. 

### 2.2. Veterinarians, Veterinary Assistants, and Clients

At the time of data collection Hong Kong had 960 registered veterinarians, where approximately 30% (280/960) were non-Chinese speakers. Until recently, Hong Kong did not have a veterinary college and attracted a large number of veterinarians from other countries (i.e., non-Chinese speakers). In the last 5 years, 44% of the veterinarians that have worked at VMC were non-Chinese speakers. Similar scenarios are found in other veterinary centres in Hong Kong. This can be extrapolated to other parts of the globe where veterinarians and clients might not speak the same language and a VAI is involved in the communication. The VAI usually also works as a veterinary nurse or technician requiring a specific skill set that involves consecutive translation or rather interpretation of verbal and written content [18]. Each clinician will regularly be supported by the same VAI to create a bond between clinician, VAI, and the client in this three-way conversation model. 

In a recent report issued by the census department of the Hong Kong SAR, 13.4% of households were reported to include domestic helpers [19]. In Hong Kong, most frequently, the caregiver is a foreign domestic helper that will accompany or bring the animal into the clinic instead of the owner. This leads to communication being held between clinician or VAI and caregiver before it is translated to the owner of the pet directly or by telephone.

### 2.3. Language

In this manuscript the authors will address communication issues when the clinician, practitioner, or veterinarian (terms used interchangeably) speaks in English and the VAI speaks in Cantonese (herewith also referred to as Chinese).

### 2.4. Cultural Competence

**Figure 2:**
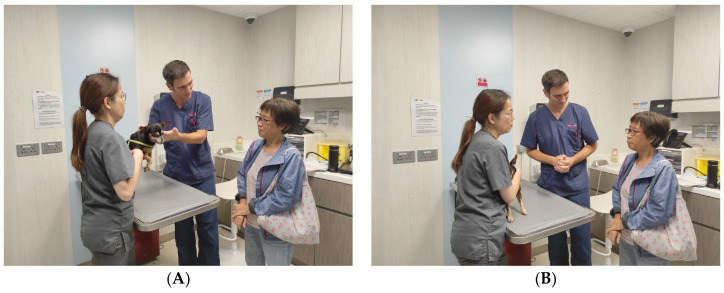
Triangular model of communication in an MCML. (**A**) Clinician talks to client in English using verbal and non-verbal communication to explain examination findings. Non-verbal communication fails as client avoids eye contact and looks at the VAI. (**B**) Here, the VAI communicates to a client in Cantonese. The clinician adopts a neutral posture while findings are explained in Cantonese.

Cultural competence is the ability to understand and respect values, beliefs, and attitudes across cultures and to respond appropriately to these differences [20]. Due to language and cultural differences in MCMLs, interactions during consultations might be minimal between clinician and client. Eye contact between veterinarian and client might be avoided, deeming non-verbal communication impossible (Figure 2). The consultation therefore might be instead conducted by a VAI that speaks Chinese in this case. As well as possessing linguistic competence, appropriate communication with the client requires for the interpreter to be able to express, explain, or paraphrase accordingly, applying both communicative competence and cultural competence [21,22]. The VAIs do not just translate in the target language (linguistic competence), but also interpret advice, persuasion, doubt, and warning, all using the corresponding linguistic expressions (communicative competence). In addition, VAIs must have socio-cultural awareness when the clinician is not familiar with the cultural setting [22]. In Hong Kong, for example, how a sick animal taken to the clinic is addressed in the communication process can provide information to the clinician. The pronoun used in Cantonese (keoi5 stands for he, she, it) will provide information about relationship between owner and animal. Cultural competence should include and address diversity in values, beliefs, and feelings [21]. The cultural background of clients should be considered by clinicians and VAIs in the communication process interpreting perception, content, and process accordingly [23,24]. Cultural competence concepts should be included in veterinary training and applied in clinical settings by incorporating it in education programs as well as continuous professional development courses. Additionally, as well as students, new graduates, and experienced clinicians, VAIs who will adopt a very important role during consultations should receive training and adhere to the same advice and guidance. 

## 3. Adaptational Differences of the Calgary–Cambridge Veterinary Consultation Guide 

A veterinary adapted version of the CCG for communication [6] is taught to undergraduates at City University of Hong Kong and is commonly used by clinicians at the veterinary teaching hospital of the same institution. The role of VAIs in this triadic communication model is paramount as they act as a projection of the attendant veterinarian for whom they are interpreting. The following section explores the CCG highlighting adaptations and recommendations with examples of these adaptations in a practical setting in this particular MCML model in Hong Kong. 

### 3.1. Preparation

The preparation of a consultation is aimed to ensure a positive and satisfactory practitioner–patient relationship. Medical records are verified as part of the preparation of the consultation. In veterinary MCMLs, some examples of the challenges we often encounter include history notes that are typed or handwritten in Chinese, other times we identify treatments, diets, and supplements recorded in the history that are traditional in Chinese culture but unknown for the non-native clinician. A VAI helps, for instance, in translating the written notes as well as explaining about the traditional alternative diet or treatment approach used by clients when preparing the consultation. Not uncommonly, customer service (CS) assistants might call clients to remind them about upcoming visits. Frequently, there are concerns or questions raised by clients before the visit that will need to be adequately translated or addressed to the non-native speaking clinician. In this instance, the VAI is required to call the client, as the clinician is usually not able to communicate without translation to clarify these concerns. 

### 3.2. Providing a Structure

An organised consultation structure using a logical flow will help keeping the consultation on time and will enhance a more efficient communication. This can be challenging in MCMLs. In general, clinicians lead the consultation, summarising after long explanations and signposting before proceeding to subsequent steps so the client can make informed decisions. In MCMLs, clients who are less comfortable communicating in English will often communicate directly with the VAI in Chinese, bypassing the clinician (Figure 2). There is a risk that an unstructured consultation will ensue, since the client has now led the narrative. The veterinarian is unlikely to know what is being communicated and will not be able to control the direction of the consultation. The clinician should remain attentive and adopt neutral body language even if not understanding what is being discussed (Figure 2). Once the subject matter becomes clear, the VAI and clinician can help to steer the conversation towards a more structured consultation.

### 3.3. Initiating the Consultation

Greetings add to first impressions. This is often when clinicians will meet a client for the first time. In our case greetings may be in English even if the client’s command of English is not fluent, with the consultation then proceeding in Chinese; however, frequently the pet is brought by a caregiver accompanying or instead of the owner [19]. Introductions of all parties are important. Addressing the clients politely by name, acknowledging their pet, knowing the name of the pet, and showing interest will help to create strong bonds. In MCMLs, greetings might be the only direct communication between client and clinician without the help of a VAI. In Hong Kong as well as in many other regions in Asia, body contact has never been common practice such as shaking hands or patting on the back. Both VAI and clinician should greet but avoid any physical contact. The recent COVID-19 pandemic has highlighted even more the avoidance of unnecessary physical contact.

The reason for the visit needs to be addressed early in the consultation. This should be clear to both clinician and VAI. Even if the reason is clear, open general questions are usually asked to allow clients to explain their concerns. Frequently in MCMLs in Hong Kong a caregiver such as a domestic helper brings the animal to the clinic. In these cases, further information might be needed from the owner through a telephonic consultation in Chinese with the help of a VAI that translates back to the clinician. All concerns, including the main reason for the consultation should be raised early in the visit so the clinician can prioritise them once they have been translated. Screening is also recommended early in the consultation to avoid delays and detect other health concerns [25,26,27].

### 3.4. Data Acquisition

Clinical records must be accurate and complete including history, examination findings, diagnostics, treatments, and future plans. Although smaller clinics in Hong Kong may record case notes by hand and/or in Chinese, it is recommended for MCMLs to use type-written English for case records. Internal memos are sometimes written in Chinese and the clinician will need to wait until the VAI translates them. Reception staff and VAIs should always write in English when communicating with non-Chinese speakers at MCMLs to avoid delays and misunderstandings. 

**Figure 3:**
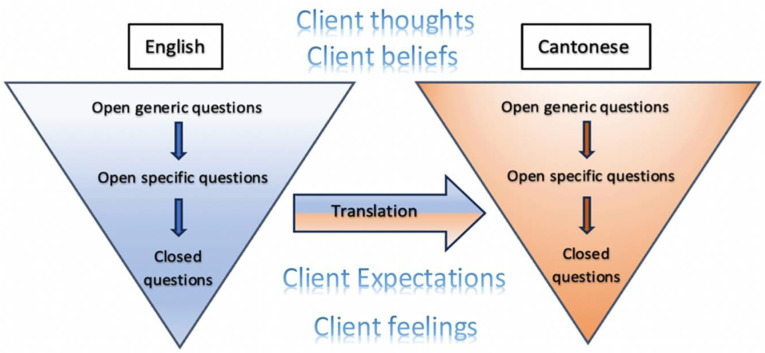
Communication flow in English and Cantonese in veterinary MCMLs in Hong Kong used for data acquisition from clients. Thoughts, beliefs, expectations, and feelings need to be addressed and accurately interpreted during consultations.

Open, followed by closed questions are used in the different stages of a consultation to continue collecting data (Figure 3). Since the VAI will be translating and conducting the conversation with the client, it is important the clinician and VAI follow the same structure during questioning (Figure 3). Data acquisition might sometimes occur through a telephonic consultation with the owner of a pet if the caregiver brought the pet. The role of the VAI is paramount as communication in these cases lacks the non-verbal component and relies solely on the VAI verbal translation. Cultural competence and linguistic competence are used by VAIs in MCMLs to interpret clients’ beliefs, thoughts, feelings, and expectations accordingly during data acquisition (Figure 3). 

#### Limiting Interruptions

Client interruptions are a common issue in medical consultations. It is reported that clients are interrupted within an average of 18 s from the start of the consultation [28]. In MCMLs, the veterinarian will need to wait for the translation from the VAI before contributing to the consultation, therefore interruptions might be less common. 

Active listening results in greater client satisfaction. When the client is not interrupted, their concerns are raised earlier. It is not unusual at MCMLs that the clinician passively listens to the VAI and the client talking in Chinese for a prolonged period. Longer conversations, however, might risk that only part of the information is translated or delivered. In these cases, a way to avoid missing information or unnecessary interruptions is promoting conversations with shorter translation periods.

### 3.5. Physical Examination

The clinician and VAI should continue gathering information, keeping an open channel of communication while conducting the physical examination of the pet. Clients often continue talking to the VAIs while the clinician examines the pet, potentially providing new and important information. When communication is not in English at MCMLs, attention should still be paid to the client to read the client’s body language and tone and perceive whether something important is being said before the VAI translates it. The body language alone accounts for 55% of the communication, whereas voice tone and verbal communication account for 38% and 7%, respectively [29]. The duration or structure of the physical examination and best practice baseline physical exam components should not differ between veterinary MCMLs and other practices [30].

### 3.6. Discussing and Sharing Information

Easily understood language should be used to not overwhelm clients. Some clients might want more detailed explanations whereas others are not interested in details. In MCMLs, the VAI will perceive if the client wants more or less detailed information regarding a particular clinical problem. Even though some clients might be fluent in English, they often prefer to have more detailed information translated into Chinese by the VAI. It is important not to overcomplicate the information given to the client or the VAI or to be excessively technical as this might trigger the client to get confused and lose interest. However, when communicating with clients who are fluent in English and have a medical background, direct communication and the use of more technical terms should be adopted. This is because on occasions, VAIs might be blamed by unhappy clients for miscommunicating what they have expressed.

#### 3.6.1. Recommending Diagnostic Treatments

Factors like demographics, background, or history might influence the way clients approach concerns. When recommendations are given to clients in Hong Kong and mainland China we need to consider factors like gender, age, whether their pets have undergone surgery previously, and whether the clients work in the field of human health, as these will influence their opinions and decisions [31]. Clients’ gender and the species being treated influence the choice of treatments such as anaesthesia as well as the way clients communicate with clinicians or VAIs [32,33]. 

Emphasis must be placed on clearly explaining why particular suggestions have been made for diagnostics, justifying the benefits and limitations of each test. Often the higher the price of a procedure, the higher the expectation from the client. Many complaints to governing bodies happen in Hong Kong due to a lack of explanation of risks or potential outcomes of particular procedures.

#### 3.6.2. Decision-Making and Understanding Recommendations

Decisions are often managed as a family in Asian cultures. That means that decisions on diagnostics or treatment are not always straightforward and are often delayed. We suggest that in MCMLs the family gathers together or that the family members not physically present are called by the VAI to make sure information is not missed or misinterpreted. Clients in Asia may not raise questions in the consultation room due to shyness or perhaps to avoid questioning experts. Recommendations in veterinary guidelines [23], suggest to asking a client to repeat what they understood, but this might come across as too direct and intimidating in Hong Kong, and because of language barriers this might trigger an embarrassing situation. Hence, we recommend that the clinician asks the VAI to repeat or explain in simpler terms what had been communicated and check with the clients if they have understood. Some clients might communicate more openly in Chinese when the non-Chinese speaking veterinarian is not present and will ask these questions to the VAIs or to someone in reception. This “time and space” should be allowed with good clinical judgement and questions should always be redirected to the VAIs or clinician. 

Compliance and concordance are important in the VCPR. Clients are more likely to adhere to plans when they participate in decision making. Client satisfaction is likely to improve compliance, and that is facilitated by shared decision-making between the clinician and the client [34]. Language barriers create a risk that decisions have been made without a true understanding. The experienced VAI should confirm that the client has understood why a treatment is necessary and what it involves.

### 3.7. Closing the Consultation

In this final part of the consultation, both veterinarian and VAI should summarise the concerns raised by the client, the different diagnostics performed or scheduled, any results of testing, and the suggested treatment, prognosis, and potential outcomes. Consent forms for procedures, if relevant, are completed in English and Chinese in MCMLs in Hong Kong, and then signed accordingly. If referral is offered, referral forms are also completed and might be given to the client or sent to the selected referral centre. Otherwise booking for revisits and future appointments are agreed in the consultation room but are completed in Chinese by reception assistants. Medication labels and prescriptions are printed in both Chinese and English and the VAIs will further explain to the client how medication is administered.

### 3.8. Building Relationships

Throughout the previous steps the clinician builds a positive rapport with the client that in the case of MCMLs is by using non-verbal communication. As well as sharing technical information, the clinician demonstrates empathy, support, understanding, and inclusion of client and animal to build up a connection. A VAI is an integral and intrinsic part of this relationship and should always be included as clients associate the clinician and the VAI as a team on which they build the relationship.

## 4. Specific Situations during Consultations

### 4.1. Consent

Consent is not simply having a client’s signature. From the veterinarian’s point of view, lack of a clear or effective explanation of a condition or treatment, misplaced assumptions of a client’s understanding, poor quality written consent forms, and excessively complicated written material for client education are potential complicating factors. These can be challenging at MCMLs where the translation and explanation from a VAI are essential. 

Verbal consent is appropriate in emergencies only, but in communities with high expectations and a high risk of complaints, entries of the agreements should always be included within medical notes. If no colleague is present, the VAI can act as a witness and entries in the records should be made accordingly.

### 4.2. Referrals

When cases are complex or beyond the clinician’s training or facilities, external or internal referral is appropriate. Respectful and positive communication between primary clinicians, clients, and specialists receiving referrals is paramount for promoting client trust and maintaining the integrity of the profession [35]. However, referral may be a sensitive issue for some clients in Hong Kong where clinician loyalty might be a barrier (as an example, one client kept a patient, unknowingly, in congestive heart failure for 2 days until their regular clinician was on duty). On the other hand, clients with financial means in Hong Kong might have higher expectations and demand referral even when unnecessary or look for 2nd, 3rd, and 4th opinions when referrals are not granted. 

### 4.3. Euthanasia

In a recent survey, 13.9% of caregivers in Hong Kong and mainland China disagreed that euthanasia is an acceptable method to end suffering in their pets when, for example, pain cannot be treated [31]. This could be due to cultural and religious beliefs, guilt, and/or societal reluctance to accept matters about death [31]. As such, there may be situations when euthanasia is recommended but not accepted by the client. In our experience, some patients with a poor quality of life get overtreated for incurable diseases until their natural death. 

The lack of understanding that euthanasia is a medical option in the face of terminal disease, despite veterinarian recommendations against expensive and complex tests or procedures, becomes an ethical issue. Clients often try alternative and/or traditional Chinese medicine if the prognosis or the outcome provided are different from the client’s unreasonable expectations. It is not uncommon for veterinarians in Hong Kong to have 4th and 5th opinion consultations for terminal patients when the client is seeking a more favourable prognosis. Veterinary boards would generally recommend presenting all available options to a client and to discuss the relative merits or drawbacks of each option so that the client can make their own decisions. Education on palliative care, end-of-life strategies and the implications for animal welfare when euthanasia is refused is required during communication with these clients. This is particularly important when euthanasia is perceived as cruel, which frequently leads to seeking consultation with another veterinarian.

## 5. Conclusions

Guidelines for consultation and communication are essential for veterinary students and practitioners, especially in the early stages of their careers. Although there is abundant literature on this topic, the communication strategies can become more challenging with cultural differences and multilinguistic scenarios where the clinician and the client communicate in different languages. 

This paper explores the challenges and adaptations encountered in multicultural and multilinguistic veterinary medical centres based on the Calgary–Cambridge Guide, including the important role of VAIs in every step of the guide in the hope that communication skills and training can be improved for more effective consultations. The authors recommend that VAIs and supporting staff at MCMLs are adequately supported and receive regular training in communication. Additionally, the authors acknowledge the need of further prospective studies that analyse and identify challenges in communication during consultations at MCMLs. These might shed light on further modifications to effectively improve communication when applying consultations guides such as the Calgary–Cambridge Guide.

## Data Availability

The data analysed for this study can be provided upon request.
